# Moral sensitivity in business: A revised measure

**DOI:** 10.1007/s12144-021-01926-x

**Published:** 2021-09-23

**Authors:** David Schmocker, Carmen Tanner, Johannes Katsarov, Markus Christen

**Affiliations:** 1 Department of Banking and Finance, University of Zurich, Plattenstrasse 32, 8032 Zurich, Switzerland; 2 Leadership Excellence Institute Zeppelin, Zeppelin University, Friedrichshafen, Germany; 3 Institute of Biomedical Ethics and History of Medicine, University of Zurich, Zurich, Switzerland; 4 Digital Society Initiative, University of Zurich, Zurich, Switzerland

**Keywords:** Moral sensitivity, Ethical sensitivity, Moral awareness, Moral competences, Moral intelligence

## Abstract

In order to manage ethical challenges in organizations and the workplace, moral sensitivity (MS)—the ability to identify and ascribe importance to moral issues when they arise in the workplace—is seen as the key prerequisite by researchers and professionals. However, despite the importance of MS, satisfactory reliable and valid measures to assess this competence are to date lacking. The present research tests the psychometric qualities of a revised MS measure for the business domain (R-MSB) that is designed to assess individual differences in moral and business-related value sensitivity. We present three different analyses with two heterogeneous samples of Swiss and German employees (total *N* = 1168). The first two studies provide good evidence of the measures’ factorial structure, its construct, and criteria-related validity. The third study examines how affective and empathic responses are associated with MS and business sensitivity (BS). The results support the view that empathic responsiveness enhances MS. The instrument’s theoretical and practical strengths, limitations, and avenues for future research are discussed.

**Supplementary Information:**

The online version contains supplementary material available at 10.1007/s12144-021-01926-x.

Recent corporate ethics scandals involving executives and employees have led to a massive loss of trust in organizations among the public and have demonstrated that ethical lapses can result in costly consequences (Agrawal & Cooper, 2017; Thomas et al., 2004). They have raised questions about how it was possible that organizations and their members could drift so far from moral common sense. In the last decade, research in business and behavioral ethics has identified numerous personal and organizational factors that facilitate illegal and unethical behavior, such as deception, stealing, fraud, or corruption (e.g., Moore & Gino, 2013, 2015; Treviño et al., 2006). One essential conclusion of this research is that even “good” people can engage in wrongdoing and even unconsciously become habituated to such behavior (Bazerman & Chugh, 2006; Bazerman & Gino, 2012; Bazerman & Tenbrunsel, 2011a; Tenbrunsel & Messick, 2004). It is argued that they do so simply because they are insensitive to ethical issues, i.e., because they are *morally blind*(e.g., Bazerman & Tenbrunsel, 2011a, 2011b; Palazzo et al., 2012; Tenbrunsel & Messick, 2004).

There is consensus that promoting *moral sensitivity*(MS) can help to alleviate moral blindness (Bazerman & Tenbrunsel, 2011b; Palazzo et al., 2012; Reynolds, 2006; Zhang et al., 2015). Generally, MS is defined as the ability to identify and to ascribe importance to moral issues when they arise in the workplace (Jordan, 2009; Karcher, 1996; Pedersen, 2009; Shaub, 1989; Sparks & Hunt, 1998). This includes, for instance, envisaging whether a course of action can violate ethical standards or codes of conduct or can harm others.

In his seminal work, Rest (1986) highlighted that MS is a necessary precursor of ethical decision-making and behavior. Without the initial recognition that moral values may be threatened, the individual sees no reason to question his/her own behavior or that of others from an ethical point of view and has no motivation to intervene (Clarkeburn, 2002; Rest, 1986; Sparks & Hunt, 1998). MS is thus considered to be a prerequisite for managing ethical challenges and ensuring moral conduct in the workplace (Jordan, 2009; Rest, 1986; Tanner & Christen, 2014). As a consequence, MS has emerged as being one key competence in many professional domains, such as business, nursing, and medicine (Lützén et al., 2006; Schluter et al., 2008).

Despite the proclaimed relevance of MS, the lack of an effective and valid measure of MS makes it difficult to advance research in this domain. Not surprisingly, several authors have therefore called for the construction and validation of a MS scale (Jordan, 2007; Miller et al., 2014). A sound MS measure would advance research in this domain, e.g., by uncovering more thoroughly individual differences and the impact of MS on decision-making and behavior. Furthermore, it could offer organizations a tool for personnel training, and evaluation of applied intervention strategies.

Thus, the purpose of this paper is to test the psychometric properties of a revised measure of MS in the business context (R-MSB), created on the basis of an earlier vignette-based measure (MSB) that has been designed to assess the sensitivity for moral values (such as fairness or respect) as well as the sensitivity for business-related values (such as profitability or performance) (the MSB is described in Schmocker et al., 2019). Below, we first describe the conceptual basis of MS and the intended improvements of the revised MSB compared to the earlier version. We then test its psychometric qualities with data from two heterogenous samples of organizational employees. Finally, we test hypothesized relationships between affective responses, empathic concern and MS.

## Conceptualizing Moral Sensitivity

The importance of MS*—*sometimes also described as ethical sensitivity or moral awareness*—*has been theorized in the business and organizational literature (for reviews on the conceptual development of MS, see Jordan, 2007; Miller et al., 2014; and Weaver, 2007). We build on the framework of other researchers who understand MS as a *social cognitive construct*(Gioia, 1992; Jordan, 2009; Narvaez & Lapsley, 2005; Reynolds, 2008). This approach conceives the accessibility and activation of moral schemas as a crucial condition for demonstrating MS, because such schemas guide attention and information processing. Schemas emerge from individual experiences and socialization rendering some more accessible than others. For example, in her pioneering work, Jordan (2009) has suggested that business schemas are more dominant than moral schemas for managers because they have much experience with business challenges, such as achieving profitability. Consistent with this, she found that, relative to academics, business managers were less likely to detect moral issues than business-related issues in ambiguous vignettes.

In line with other researchers, we consider MS as an individual’s ability to identify and to ascribe importance to moral issues when they arise in the workplace (Jordan, 2009; Karcher, 1996; Pedersen, 2009; Shaub, 1989; Sparks & Hunt, 1998). The ascription of importance in the above definition is crucial because without assigning some priority to moral issues, they are likely to be filtered out in the subsequent decision-making process (e.g., Miller et al., 2014). Building on this definition, a financial advisor, for example, is considered morally sensitive if he/she is aware that lying to the customer would violate some ethical standards, and that it is important to consider this aspect in interactions with customers.

Expanding on our earlier work that has concentrated more on cognitive processes, we stress here that MS may also draw on *affective reactions*. Building on Rest’s (1986) original work whereby MS includes both cognitive and affective recognition, some conceptions of MS highlight that affective responses are preconditions of MS. As Clarkeburn argued, “there can be a strong affective response before extensive cognitive coding” (2002, p. 441).

This reasoning concurs with the prevalence of dual process models which account for the fact that human information processing not only involves cognitive, deliberate, slow, and effortful operations (System 2) but also automatic, intuitive, fast, and often emotionally charged operations (System 1) (e.g., Epstein, 1991; Kahneman, 2011). Similarly, in the field of moral psychology, Haidt (2001) proposed a moral intuitionist model whereby quick and automatic affect-laden judgments (“gut feelings”) often precede moral reasoning. Haidt and others share the view that affect-laden responses to situations are important cues for judgment (“affect as information”) (Damasio, 1994; Schwarz & Clore, 1983). It has been stated that morally charged affective responses, such as moral outrage, anger, guilt or contempt reflect an inherent “moral sense” (Prinz, 2014) or signal the violation of essential values to individuals (Hanselmann & Tanner, 2008; Tetlock et al., 2000).

Beyond affective responses, it is plausible to assume that MS may also benefit from interpersonal skills such as empathy. *Empathy—*including empathic concern and perspective-taking—enables us to understand and feel the emotional state of others and to imagine whether others might be affected by one’s action (e.g., Davis, 1980; Eisenberg, 2000; Hoffman, 2000), thereby cultivating a sense of connection to others (Tirri & Nokelainen, 2011). The importance of empathy for MS seems obvious: When people realize that others may suffer from their actions, they are more likely to recognize that a moral issue is at stake (Narvaez, 2010; Tanner & Christen, 2014; Tenbrunsel & Smith-Crowe, 2008).

Therefore, the second aim of this research is to offer a preliminary insight into the role of affective and empathic responsiveness for MS and business sensitivity (BS). We expect that both the spontaneous apprehension of moral standards being violated or the comprehension of another’s state will ease MS (rather than BS, or even hinder BS). Again, using the example of a financial advisor, it is suggested that such a person would be more likely to recognize moral issues and ascribe importance to them if he/she would feel outrage when seeing other financial advisors lying to customers and/or if he/she could imagine how a customer would feel when the customer determines that he/she was deceived.

## MS Measurements (in the Business Context)

While psychometrically sound MS measures are lacking, preliminary efforts to assess MS have at least two important features in common: They are usually domain-specific to better account for ethical challenges that are typical for particular areas of practice (e.g., workplace, medicine). Furthermore, they generally rely on some type of scenarios (also called “vignettes”) to which participants attribute issues they consider as involved in the situation (for excellent overviews of methods to assess MS, see Jordan, 2007, Miller et al., 2014).

While our measure is also domain-specific and vignette-based, it differs from other approaches in some noteworthy ways. To obtain a valid measurement, one initial challenge is to prevent making it obvious that the situational description contains moral dimensions. Some approaches assess MS by asking participants whether the described scenario contains an ethical issue or by using “ethical” or “moral” in the wording of items (e.g., Reynolds, 2008). However, this can alert the participant to consider moral aspects (Tenbrunsel & Smith-Crowe, 2008). Our measure addresses this concern by avoiding such wording. Furthermore, our instrument represents a quantitative approach, while prior MS measures in the business context often rely on open-ended questions (see e.g., Butterfield et al., 2000; Erwin, 2000; Jordan, 2007). However, the most notably distinctive feature of our approach is that our measure is designed to assess both sensitivity for moral values *and* sensitivity for business values. We deem it relevant to account for the fact that not only sensitivity for moral issues would be desirable, a responsiveness to business values is also indispensable and legitimate for business professionals.

### The Earlier Version of MS and BS Measure (MSB)

Details about our measure to assess MS and BS and its validation are described in Schmocker et al. (2019). The procedure was as follows: Participants were asked to imagine being a member of a company’s task force. In this function, they were faced with current problems of the organization (vignettes) and asked to report those aspects that might be relevant to solve the problem. Those vignettes were built in collaboration with practitioners to ensure that they reflect realistic problems in an organization that not only tap into economic aspects but also put ethical values at risk (such as fairness, respect, no harm, loyalty), and which involve different stakeholders (e.g., exchanges with employees, customers, other organizations). After each vignette, participants were presented with different statements (representing moral or business-related issues) and asked to perform two tasks: a *selection* and a *weighing* task. In the selection task, they were required to indicate (with *yes* or *no*) whether they consider each issue to be (un)related to the situation described. In the subsequent weighing task, only the statements that participants had chosen in the first step were presented again. Participants were asked to indicate the importance of each statement by distributing 10 points to the remaining statements. The corresponding statements were developed and rigorously tested in previous studies to conceptually correspond either with moral or business-related issues (Schmocker et al., 2019).

Building upon these two tasks*—*the number of moral or business-related statements selected in the first step (reflecting the ability to recognize moral and business-related issues) and the points people assigned to the selected statements in the weighing task (representing the ascription of importance)*—*the individual’s scores for MS and BS was calculated.

As indicated, prior to the construction of this instrument, extensive studies and analyses were carried out to identify core moral and business values, to develop appropriate value statements, and realistic and comprehensive vignettes (for a detailed description of these steps, see Christen et al., 2014; Ineichen et al., 2017; Schmocker et al., 2019). Overall, these studies resulted in six vignettes with corresponding value statements.

### The Revised Version of MS and BS Measure (R-MSB)

Although we found good evidence for the reliability and validity of the MSB (Schmocker et al., 2019), we identified two major problems that led us to make an adjustment. The first problem is related to the weighing task that asked people to distribute a total of 10 points to the selected statements. The nature of this task excludes the possibility that an individual can rank both the moral *and*business-related values as equally important (i.e., only 10 points could be distributed overall, and this may require trade-off decisions). However, we believe that MS and BS do not necessarily have to be mutually exclusive; an individual may be highly sensitive to *both* moral and business values.

The second problem is of a statistical nature. The two-fold procedure that differentiates between the selection and weighing tasks excludes the possibility of conducting exploratory and confirmatory factor analyses (EFA, CFA). However, such statistical procedures are common and valuable to examine the measure’s construct validity and to consider item reduction.

To overcome these problems, we adapted the MSB as follows: First, we combined the selection and weighing task by directly asking people to rate the importance of considering a particular issue when deciding which action to take. Note that both elements of MS identifying an issue *and* ascribing importance to it, are still included in this adapted procedure. One issue that is not seen as related to a current situation is unlikely to be rated as important. However, if an issue is recognized, then it can be considered as more or less important. In doing so, MS and BS can be assessed by calculating the means over the ratings to the value statements. This, in turn, allows to conduct factor analyses. As part of this adaption, we slightly rephrased the value statements. Second, while including all six vignettes from the first version, we wished to examine the usefulness of five additional vignettes. Third, we further shortened the vignettes from 150 words to 100 words. Next, we present research to test this revised version (R-MSB).

## Overview of Samples and Studies

Using online surveys, data from two independent and heterogeneous samples of German and Swiss employees were collected to assess the validity of the R-MSB and to examine potential predictors of MS [BS]. To attain broad samples of participants working in various industries, participants were recruited via panels of a market research agency.

*Sample A* consisted of 651 German and Swiss employees. Another 179 participants were excluded due to a lack of variation in their answering patterns (straight-line responses) or questionable participation times (participants who completed the survey in less than half of the median processing time). The inclusion criteria included being at least 20 years of age, working full-time or part-time, living in Germany or Switzerland, and being fluent in German. The sample was balanced for German and Swiss respondents and gender. The mean age was 43.76 years (*SD* = 11.61; age range: 20–68 years).

*Sample B* consisted of 599 German and Swiss employees, and another 149 participants were excluded based on the above-mentioned criteria. Participants fulfilled the same requirements as participants from sample A. Again, the sample was balanced for country of residence and gender. The mean age was 43.84 years (*SD* = 11.35; age range: 20–64 years).

Table 1 reports further education- and work-related characteristics of the samples.

**Table 1:** Further descriptive characteristics of samples

	Sample A (N = 651)	Sample B (N = 599)
Education		
Apprenticeship	36.1%	46.9%
Secondary education	15.1%	22.4%
Higher education	44.5%	27.9%
Other	4.3%	2.8%
Employment		
Full time	72.8%	76.5%
Part time	27.2%	23.5%
Tenure		
1–5 years	7.7%	9.2%
6–10 years	13.7%	12.4%
11–20 years	26.7%	25.0%
< 20 years	51.9%	53.4%
Economic Sector		
Industry, trading or construction	32.3%	33.1%
Education, health or social services	23.7%	20.5%
Administration and services	23.5%	25.4%
Others	20.5%	21.0%

Study 1 was designed to assess the underlying factorial structure of the different value statements and to confirm the robustness of the proposed structure performing EFA and CFA. The goal of Study 2a was to provide further evidence of the R-MSB’s convergent and discriminant validity by comparing the measure with theoretically (un)related constructs (using data from sample B). Study 2b further examined its criterion-related validity by conducting a group comparison among selected participants from samples A and B. Finally, building on sample B, the goal of Study 3 was to take initial steps to explore predictors of MS [BS] by testing the relationships between affective responses, empathic concern, and MS [BS] using structural equation models (SEM). Data were analyzed using either SPSS (version 25) or open source software R.

## Study 1: Exploratory and Confirmatory Factor Analyses

EFA and CFAs were performed using data from sample A (*N* = 651) and sample B (*N* = 599) to examine the factorial structure of the R-MSB. Our first hypothesis was to identify a two-dimensional structure with MS and BS representing two distinct factors (H1). Sample sizes were considered to be good to excellent for factorial analyses suggesting two factors (Mundfrom et al., 2005).

### Method

#### Procedure and Measure

As in the earlier version, participants were asked to imagine being on a company’s task force addressing several organizational problems (= vignettes). They were asked to indicate which issues (= statements) they would consider more or less important when deciding what action to take. Overall, we had 11 vignettes. In sample A, we created two sets of six vignettes to avoid excessive burden for the participants (for comparative reasons, Vignette 1 was provided to both groups). Participants were randomly assigned to one of the sets, and vignettes were presented in a randomized order. Sample B was only provided with the six vignettes (in a randomized order) that remained after the EFA. After each vignette, participants received a total of eight statements (in randomized order) related to the described situation.

As in the MSB, the statements were designed to reflect four typical moral values (fairness, loyalty, non-maleficence, and respect) and four typical business values (profitability, performance, competition, and reputation). Specifically, participants read “How important do you find to consider the following statements for the pending decision?” They then rated all eight statements on a seven-point scale (1 = *not important at all*, 7 = *very important).* This procedure was repeated for each vignette (for an example of a vignette with the corresponding value statements, see Appendix).

### Results

#### Exploratory Factor Analyses

In the first step, for each of the eleven vignettes (sample A), the four items related to moral values (MS) and the four statements related to business values (BS) were subjected to an EFA (principal axis factoring, promax oblique rotation) (Fabrigar et al., 1999). These analyses revealed a clean structure of two factors for six vignettes (eigenvalues >1) with the four MS and four BS items clearly loading on the corresponding factor (loadings of > .45) and cross-loadings clearly lower than our cut-off of .30. The other five vignettes were excluded from further analyses due to lower factor loadings and cross-loadings exceeding .30.

The EFA was rerun over all remaining six vignettes with the data from sample A. To do this, all value statements reflecting the same moral or business value were averaged across these vignettes. The resulting means were subjected to the EFA. This analysis resulted in two factors (eigenvalues >1) with the MS and BS statements clearly loading on either the MS and BS factor (loadings of ≥. 70); cross-loadings were clearly lower than .30. These two factors accounted for 79.8% of the variance. Factor loadings are reported in Table 2.

**Table 2:** Factor loadings of EFA and CFAs (Study 1)

		EFA 1		CFA 1	CFA 2
	Value	(1)	(2)	(*λ*)	(*λ*)
(1) MS component	Fairness	−.04	**.88**	.87	.91
	Loyalty	.17	**.73**	.73	.81
	Non-maleficence	−.04	**.89**	.88	.90
	Respect	−.06	**.86**	.85	.88
(2) BS component	Profitability	**.91**	−.12	.88	.87
	Performance	**.87**	.01	.87	.92
	Competition	**.92**	−.03	.87	.97
	Reputation	**.70**	.20	.71	.73
Eigenvalue		4.01	2.38		
% variance explained		50.14	29.69		

Boldface indicates the main loading in the EFA that was run over the six remaining vignettes (sample A, *N* = 651) column. Only the main loadings of the final Model 3 are reported in the CFA 1 (sample A) and CFA 2 (sample B, *N* = 599) columns (cross-loadings were > .28)

Table 3 reports the means, standard deviations, internal consistencies (Cronbach’s alpha), and intercorrelations among the two subscales. Both scales revealed high levels of internal consistency (*α*s = .91). Despite the analyses revealing two distinct factors, we note that MS and BS are moderately but positively correlated, indicating that MS and BS are not opposed dimensions. This suggests that being sensitive to business issues does not necessarily preclude being sensitive to moral issues and vice versa.

**Table 3:** Means, standard deviations, internal consistencies and intercorrelations (Study 1)

		*M*	*SD*	*α*s	*r*
Sample A *(N* = 651)	MS component	5.13	.86	.91	
	BS component	4.14	.87	.91	.27**
Sample B (N = 599)	MS component	5.06	.88	.93	
	BS component	4.60	.95	.94	.31**

**p < .01

#### Confirmatory Factor Analyses

CFAs were conducted with data from sample A and B to test the robustness of the dimensionality across the remaining six vignettes (using lavaan package in R software)*.* Three measurement models were estimated: Model 1 proposed all eight value statements loading on one factor; Model 2 proposed an MS and BS factor; and Model 3 also proposed an MS and BS factor but allowed two cross-loadings.

Examination of the loadings in the CFAs revealed that the MS value statement reflecting the value loyalty also loaded on the BS factor. The BS value statement reflecting the value reputation also loaded on the MS factor (see Table 2). We therefore allowed those two cross-loadings which clearly improved the fit indices. Allowing these cross-loadings between some MS and BS items is also theoretically meaningful. For example, though reputation is primarily considered a business-related value, it is not surprising that it can have various other subordinated connotations. While some may see having a good name and standing mainly as an asset for economic success, others may understand that reputation arises from signaling compliance with ethical standards (Fombrun & Shanley, 1990). Similarly, while loyalty is usually seen as reflecting a fundamental moral value (Haidt & Joseph, 2004; Waytz et al., 2013) that places priority to solidarity with the group, others may see employees acting in the service of a team or the organization as supporting the organization’s functionality and competitiveness (Waytz et al., 2013).

Table 4 reports the fit indices for each model. We used the comparative fit index (CFI), the Tucker-Lewis-Index (TLI), the root mean square error of approximation (RMSEA), the standardized root mean square residual (SRMR), the Akaike information criterion (AIC), and the Bayesian information criterion (BIC) to assess the model fit. Though researchers differ in their recommendations when evaluating the model fit, CFI and TLI values above .90 are usually considered as acceptable. Values above .95 indicate a good model fit (MacCallum et al., 1996). RMSEA values between 0.08–0.10 and SRMR values equal or lower than 0.08 indicate an acceptable fit (MacCallum et al., 1996). As to the AIC and BIC, smaller values indicate a better model fit. Regarding AIC, a difference of 2 means that both models fit essentially equally good, a difference of 5 means that the model with the lower AIC fits a bit better, a difference of 10 is pretty strong evidence that the model with the lower AIC fits better (Burnham & Anderson, 2004).

**Table 4:** Fit indices for various CFA models (Study 1)

Model	*χ*^*2*^*(df)*	CFI	TLI	RMSEA	SRMR	AIC	BIC	Model comparisons^a^ ∆χ^2^ (df)
Sample A (N = 651)								
M1: Single-factor model	1513.26 (20)	.40	.16	.34	.27	13,442.66	13,514.32	M1 vs. M3: 1232.30 (3)*** M2 vs. M3: 74.57 (2)***
M2: Two-factor model	165.76 (19)	.94	.91	.11	.09	11,730.14	11,806.28	
M3: Two-factor model with cross-loadings	72.92 (17)	.98	.96	.07	.03	11,612.65	11,697.74	
Sample B (N = 599)								
M1: Single-factor model	1925.89 (20)	.34	.08	.40	.29	11,541.40	11,611.72	M1 vs. M3: 4523.30 (3)*** M2 vs. M3: 146.78 (2)***
M2: Two-factor model	249.37 (19)	.92	.88	.14	.10	9444.31	9519.03	
M3: Two-factor model with cross-loadings	132.34 (17)	.96	.93	.11	.05	9293.28	9376.79	

We considered the following indices as indicating acceptable fit: CFI and TLI > .90, RMSEA between 0.08–0.10, and SRMR equal or lower than 0.08 (Hu & Bentler, 1999; MacCallum et al., 1996). As to AIC and BIC, smaller values indicate a better model fit (Burnham & Anderson, 2004)

^a^Satorra-Bentler scaled χ^2^ difference test, *** *p* < .001

As Table 4 shows, the one-factor model (Model 1) revealed poor fit indices. Model 2, proposing a simple two-factor model, produced acceptable CFI and TLI fit indices. However, RMSEA and SRMR fell outside of the recommended standards. Model 3 produced good or acceptable fit indices indicating a suitable data fit. Comparing the latter model with the two other models, the smaller AIC and BIC values for Model 3 indicate a better fit. Another comparison, using the Satorra-Bentler scaled χ^2^ difference test confirmed that Model 3 fits significantly better than Model 1 or 2 (see Table 2 for the standardized main-factor loadings of Model 3). That is, the findings support H1, a two-factor structure of the R-MSB.

## Study 2a: Convergent and Discriminant Validity

Data from sample B were used to examine the convergent and discriminant validity of the R-MSB by comparing this measure with other theoretically related and unrelated constructs. Specifically, to test convergent validity of each of the two factors, we examined the average variance extracted (AVE) in the CFA. To test the discriminant validity, we used the square root of the AVE scores and compared these values with the correlation coefficients of the MS [BS] latent constructs and other latent constructs.

We further examined bivariate correlations between the MS [BS] components and the other constructs. Regarding the MS component, we expected moderate but positive relations with following concepts: moral attentiveness (H2a), moral intuitions (H2b), empathy (H2c), and communal values (H2d). *Moral attentiveness*(Reynolds, 2008) is defined as the extent to which individuals actively and chronically search for moral aspects in daily life. MS is somewhat different in the sense that it builds on a context-specific approach, while Reynold’s concept of moral attentiveness is more trait-like and abstracting from particular situations. According to Haidt (2001), *moral intuitions* refer to “the sudden appearance in consciousness of a moral judgement” (p. 818). *Empathy* can roughly be defined as the individual’s responsiveness to other people (Davis, 1980; Leibetseder et al., 2016). *Communal values*(Trapnell & Paulhus, 2012) reflect people’s striving for bonding social relationships. These concepts share the appreciation of moral and prosocial values with MS.

For the BS component, we hypothesized moderate but positive relations with two concepts: Machiavellianism (H3a) and agentic values (H3b). *Machiavellianism* refers to calculated manipulation to achieve personal goals while disregarding moral issues (Ulbrich-Herrmann, 2014). *Agentic values* represent people’s striving for self-advancement in social hierarchies, e.g., by focusing on power and economic success (Trapnell & Paulhus, 2012). We assumed that these concepts share the appreciation of business-oriented values with BS.

Finally, we expected both MS and BS to be unrelated to *social desirability* (H4), the tendency to attribute socially desirable values and characteristics to oneself and to reject the non-desirable ones (Helmes & Holden, 2003). With our measure, we intend to assess MS and BS independently of social expectations and norms. Therefore, ideally, no connection between these constructs should exist.

### Method

#### Procedure

As mentioned above, participants in sample B worked through six vignettes to assess MS and BS. After each vignette, participants of this sample, were provided with some items to assess affective and empathic responsiveness (will be relevant later, see Study 3), and then the MS and BS statements. After this step, participants were provided with other established measures to assess theoretically related concepts. To avoid excessive burden, participants were only questioned on two of the six related concepts. They were randomly assigned to one of three sets of questionnaires. Hence, sample B consisted of three subsamples: B1, B2, and B3.

*Measures of related concepts.*

##### Moral Attentiveness

A German version of the moral attentiveness scale from Reynolds (2008) was administered to assess the extent to which individuals actively and chronically search for moral aspects in their daily life (Pohling et al., 2014). The measure consists of the perceptual moral attentiveness subscale (7 items; e.g., “On a typical day, I face several ethical dilemmas”, *α* = .90) and the reflective moral attentiveness subscale (5 items; e.g., “I regularly think about the ethical implications of my decisions”, *α* = .90), using a seven-point scale (1 = *strongly disagree*, 7 = *strongly agree*).

##### Moral Intuitions

The Moral Foundations Questionnaire by Graham et al. (2011) was designed to assess five different patterns of moral intuitions with six items each. For each pattern, two types of items were answered, using two different seven-point scales (1 = *not very relevant,* 7 = *very relevant* or 1 = *strongly disagree,* 7 = *strongly agree*). The five intuitions are care/harm (“Whether or not someone suffered emotionally” or “One of the worst things a person could do is hurt a defenseless animal”, *α* = .78), fairness/reciprocity (“Whether or not some people were treated differently” or “Justice is the most important requirements for a society”, *α* = .78), ingroup/loyalty (“Whether or not someone did something to betray his or her group” or “It is more important to be a team player than to express oneself”, *α* = .53), authority/respect (“Whether or not someone showed a lack of respect for authority” or “Respect for authority is something all children need to learn”, *α* = .62), purity/sanctity (“Whether or not someone violated standards of purity and decency” or “Chastity is an important and valuable virtue”, *α* = .61). We used a German version of the MFQ (Moral Foundations, 2013).

##### Empathy

Empathy as measured using the German Empathy scale (E-Scale after Leibetseder et al., 2016). The 21-item questionnaire builds on prior empathy instruments (e.g., Davis,1980; and Mehrabian & Epstein, 1972). It assesses a general empathy factor with items that stem from four empathy dimensions (*α* = .92), namely cognitive sensitivity for fictitious situations (“When I hear an interesting story, I often imagine how I would feel about it”), emotional sensitivity for fictitious situations (“I can very easily feel the feelings of novel characters”), emotional correspondence in real situations (“The people around me have a big influence on my mood”), and cognitive correspondence in real situations (“I tend to get caught up in a friend’s problems”). Responses were given on a seven-point scale (1 = *does not apply at all*, 7 = *applies strongly*).

##### Agentic and Communal Values

These values were assessed with the short questionnaire by Trapnell and Paulhus (2012). The 12 items were translated into German using the backtranslation method (Brislin, 1976). To assess individuals’ preference for agentic or communal values, people were provided with a list of values, and each value was accompanied by some specific characteristics in parentheses to clarify the meaning of the value. Agentic values incorporate values, such as achievement or power, while communal values encompass values, such as honesty or forgiveness. Each value category was represented by six items. An item example for an agentic value is “POWER (control over others, dominance)” (*α* = .80), and an item example for a communal value is “HONESTY (being genuine, sincere)” (*α* = .83). Items were rated on a seven-point scale (1 = *not important to me*, 7 = *highly important to me*).

##### Machiavellianism

People’s propensity toward Machiavellianism was assessed with a scale by Ulbrich-Herrmann(2014). The 14 items (e.g., “Modesty is not only useless but also harmful”, *α* = .89) were rated on a seven-point scale (1 = *completely wrong*, 7 = *absolutely true*).

##### Social Desirability

Was assessed used a German 6-item scale by Kemper et al. (2014), which builds on other common social desirability scales, such as the SES-17 scale (Stöber, 1999) or the SDS-CM scale (Crowne & Marlowe, 1960). It encompasses two subscales: individual’s tendency to exaggerate positive personal qualities (3 items; *α* = .67) and individual’s tendency to understate negative qualities (3 items, *α* = .66). Item examples are “In disputes I always remain factual and objective” or “It has happened before that I took advantage of someone”. Responses were on a seven-point scale (1 = *does not apply at all,* 7 = *applies strongly*).

### Results

To examine convergent validity, we tested the average variance extracted (AVE) in a measurement model that includes MS [BS] as well as other theoretically related constructs. Table 5 shows that the AVE for MS and BS clearly exceeds the conventional threshold of .50 (Fornell & Larcker, 1981) supporting good convergent validity. To examine discriminant validity, we compared the square root of the AVE scores for MS [BS] with the correlations between the MS [BS] latent construct and other latent constructs. Satisfactory discriminant validity is given when the square root of the AVE scores for MS [BS] is greater than the correlations between MS [BS] and the other constructs (Fornell & Larcker, 1981; Hair et al., 2014). Table 5 (results for subsamples B1, B2, and B3) shows that the correlation between MS [BS] and any other construct was smaller than square root of the AVE scores for MS [BS]. This confirms that the MS [BS] measure has good discriminant validity.

**Table 5:** Confirmatory factor analyses comparing the R-MSB measure to theoretically relevant constructs (Study 2)

Constructs	M	SD	AVE	1	2	3	4	5	6	7	8	9
Sample B1 (n = 201)												
1. MS (R-MSB measure)	5.03	0.86	0.78	**(0.88)**	.37^**^	.46^**^	−.27^**^					
2. BS (R-MSB measure)	4.60	0.93	0.78	0.32	**(0.88)**	.07	.12^t^					
3. Empathy (E-Scale)	4.67	0.94	0.59	0.45	0.08	**(0.77)**	−.27^**^					
4. Machiavellism	3.22	1.06	0.38	−0.33	0.16	−0.25	**(0.62)**					
Sample B2 (n = 196)												
1. MS (R-MSB measure)	5.11	0.86	0.79	**(0.89)**	.34^**^	.01	.38^**^	−.02	.07			
2. BS (R-MSB measure)	4.66	1.00	0.82	0.32	**(0.91)**	.31^**^	−.01	−.11	−.06			
3. Agentic Values (ACV)	5.85	0.79	0.42	0.44	−0.04	**(0.64)**	.09	.22^**^	.11			
4. Communal Values (ACV)	4.24	1.08	0.46	−0.07	0.31	0.01	**(0.68)**	.04	.10			
5. Perceived Moral Attentiveness (MA)	3.29	1.32	0.58	−0.02	−0.11	0.06	0.25	**(0.76)**	.79^**^			
6. Reflected Moral Attentiveness (MA)	3.65	1.44	0.64	0.07	−0.04	0.09	0.12	0.88	**(0.80)**			
Sample B3 (n = 202)												
1. MS (R-MSB measure)	5.03	0.88	0.78	**(0.88)**	.20^**^	.63^**^	.62^**^	.24^**^	.24^**^	.28^**^	.24^**^	−.20^**^
2. BS (R-MSB measure)	4.55	0.93	0.79	0.14	**(0.90)**	.06	.00	.14^*^	.33^**^	.09	.17^*^	.00
3. Harm (MFQ)	5.66	0.94	0.40	0.73	0.01	**(0.63)**	.79^**^	.27^**^	.21^**^	.39^**^	.24^**^	−.25^**^
4. Fairness (MFQ)	5.54	0.91	0.45	0.70	−0.03	0.98	**(0.67)**	.28^**^	.26^**^	.33^**^	.19^**^	−.16^*^
5. Ingroup (MFQ)	4.54	0.83	0.19	0.45	0.21	0.55	0.56	**(0.44)**	.51^**^	.57^**^	.26^**^	−.08
6. Authority (MFQ)	4.69	0.90	0.23	0.30	0.40	0.43	0.40	0.86	**(0.48)**	.48^**^	.21^**^	−.02
7. Purity (MFQ)	4.21	0.99	0.21	0.42	0.11	0.66	0.54	0.88	0.84	**(0.46)**	.13^t^	−.08
8. Positive Social Desirability (KSE-G)	4.73	1.04	0.41	0.27	0.18	0.30	0.24	0.35	0.38	0.31	**(0.64)**	−.25^**^
9. Negative Social Desirability (KSE-G)	3.17	1.36	0.42	−0.22	0.04	−0.33	−0.27	−0.13	−0.04	−0.20	−0.37	**(0.65)**

Values on the diagonal (bold, in parentheses) are square root of AVE; values above diagonal are bivariate correlations; values below diagonal are latent construct correlations

*RS* real situation; *FS* fictious situation; *ACV* Agentic and Communal Values; *MA* Moral Attentiveness; *MFQ* Moral Foundation Questionnaire; *KSE-G* short scale for social desirability

^t^*p* < .10, * *p* < .05, ** *p* < .01

We then examined the bivariate correlations between MS [BS] and the other constructs to study the soundness of the conceptual specification of MS [BS] (see also Table 5). In line with H2b, MS correlates positively with all moral intuition components (*rs* ranging between .24 and .63, *ps* <. 01). Referring to Cohen’s conventions to interpret effect sizes, these correlations represent small to large effects (Cohen, 1988). We also found significant, positive correlations between MS and the harm and fairness intuitions (*rs* higher than .60, *ps* <. 01), representing large effects. They reflect the common appreciation of the values of fairness and non-maleficence inherent to both instruments. Supporting H2c and H2d, MS is also positively related to the empathy scale (*r* = .46, *p* <. 01) and communal values (*r* = .38, *p* < .01), both representing moderate effects. In contrast to our expectations (H2a), we found no significant correlations between MS and the moral attentiveness subscales. This may derive from the fact that, compared to our context-specific measure, moral attentiveness is assessed on a rather abstract and context-unrelated level (Pohling et al., 2014; Reynolds, 2008).

Furthermore, as hypothesized in H3a and H3b, we found a marginally significant positive correlation between BS and Machiavellism (*r* = .12, *p* < .10), representing a small effect and a significant correlation between BS and agentic values (*r* = .31, *p* < .01), representing a moderate effect. Different from our expectations (H4), MS (and partially BS) was significantly related to the positive and negative social desirability subscales (*rs* varying between −.20 and .24, *ps* <. 05), both representing small effects. This may imply that our measure is not free from biased response styles. Yet, as will be discussed later, there is also recent research contrasting this response style perspective with the alternative proposition that social desirability scales may reflect truly socially desirable traits rather than biased responses (de Vries et al., 2014; Uziel, 2010).

Overall, the findings and patterns of correlations, mostly provide good support for the convergent and discriminant validity of our instrument.

## Study 2b: Criterion-Related Validity

Supporting the criterion-related validity of our former measure, the MSB, we found that employees of non-governmental organizations (NGOs) yielded higher MS scores than business managers/bankers, whereas no differences were found on the BS dimensions (Schmocker et al., 2019). In a similar vein, we wished to compare two contrasting groups, employing the R-MSB. Drawing on the data from samples A and B, we compared employees from the trading, industry, and construction sectors (*n*_*1*_ = 408) with employees from the educational, health, or social sectors (*n*_*2*_ = 277).

We hypothesized (H5a) that the latter group would be more sensitive to moral issues because these individuals do more directly and routinely face problems that challenge ethical issues, such as fairness, human rights, caring, or harming others. As an outcome of their socialization, employees from the educational, health, or social sectors should therefore have moral schema more accessible than employees from the other three sectors. In contrast, we expected (H5b) that the two groups would not differ in their sensitivity towards business issues because both groups are likely to face organizational and financial challenges.

### Results

Preliminary analyses of the data confirmed normal distribution, and thus we conducted *t*-tests for the group comparisons. Supporting H5a, the group working in the educational, health, or social sector demonstrated significantly higher MS scores (*M* = 5.21, *SD* = .84) than the employees working in the trading, industry or construction sector (*M* = 4.99, *SD* = .82; one-tailed*t*-test, *t*(683) = 3.42, *p* < .001, Cohen’s *d* = .27). In line with H5b, the educational, health, or social sector (*M* = 4.66, *SD* = 1.05) did not differ from the trading, industry or building sector (*M* = 4.66, *SD* = .86) in their sensitivity for business-related issues (two-tailed *t*-test, *t*(513) = 0.03, *p* = .977, Cohen’s *d* = .00). These results confirm H5a and H5b. The capability of the instrument to differentiate between those groups in terms of MS (which was of main interest) supports the measure’s criterion validity.

## Study 3: Examining Predictors of MS

We further tested the predictive relationships between affective responses, empathic concern, and MS [BS]. Building on data from sample B (*N* = 599), we hypothesized that both affective responses (H6a) and empathic concern (H6b) are positively linked to MS, but have no or even a negative relationship with BS.

### Measures

Affective responses and empathic concern were assessed directly after each vignette (and prior to the MS and BS statements). The items were adjusted to the specific content of each vignette (see Appendix). To assess *affective responses,* and drawing on previous scales (Tanner et al., 2009; Tetlock et al., 2000), participants rated the extent to which they would perceive the decision made in the corresponding vignette as outrageous, shameful, acceptable, or praiseworthy (4 items, the latter two items reverse-coded). For example, regarding a situation in which the son of a client has applied belatedly for a job position in the company, participants were asked “To which extent would you judge it as outrageous if the client’s son had been chosen for the position?”, on a seven-point scale (1 = *not at all*, 7 = *very much*). Items were averaged across all six vignettes (*α* = .87).

To assess *empathic concern*, we adapted three items of the German Saarbrücken Personality scale (Paulus, 2009). Participants rated the extent to which some statements would apply to the described vignette on a seven-point scale (1 = *does not apply at all*, 7 = *applies very much*). For example, referring to the nepotism vignette, participants were asked to rate the item “Seeing how someone might be hired because of personal contacts, makes me want to protect other applicants”. Again, items were averaged across all six vignettes (*α* = .91). Finally, serving as two filler items within this scale, participants were also asked to rate the decision’s acceptability as a business practice.

### Results

Preliminary analysis across all vignettes (EFA) confirmed that affective, empathic responsiveness, MS and BS represented four distinct factors. Employing structural equation models (SEM) was then performed with R (using lavaan package), with maximum likelihood as the method of estimation, to examine the impact of affective responses and empathic concern on MS [BS]. We compared three alternative structural models to assess the proposed relationship. In line with H6a and H6b, Model 1 specified for each, affective responses and empathic concern, direct links to MS and BS; Model 2 specified an indirect relationship between affective reactions on MS and BS through empathic concern; and Model 3 examined an indirect relationship between empathic concern on MS and BS through affective responses. A Satorra-Bentler scaled χ^2^ difference test compared the models and revealed that Model 1 provided the best data fit (see Table 6).

**Table 6:** Fit Indices for Various SEM Models (Study 3)

Model	*χ*^*2*^*(df)*	CFI	TLI	RMSEA	SRMR	AIC	BIC	Model comparisons^a^ ∆χ^2^ (df)
M1: Direct effects on MS and BS	511.11 (82)	.93	.90	.10	.07	17,605.41	17,838.35	M1 vs. M2: 14.32 (2)*** M1 vs. M3: 183.20 (2)***
M2: Indirect effect of affective responses	565.53 (84)	.92	.90	.10	.08	17,620.73	17,844.89	
M3: Indirect effect of empathic concern	755.48 (84)	.89	.87	.12	.13	17,852.67	18,076.83	

*N* = 599. We considered the following indices as indicating acceptable fit: CFI and TLI > .90, RMSEA between 0.08–0.10, and SRMR equal or lower than 0.08 (Hu & Bentler, 1999; MacCallum et al., 1996). As to AIC and BIC, smaller values indicate a better model fit (Burnham & Anderson, 2004). A direct comparison between Model 2 and 3 was not possible due to the same degrees of freedom. However, the fit indices indicate a better fit for Model 1

^a^Satorra-Bentler-scaled χ^2^ difference test, *** *p* < .001

**Fig. 1:**
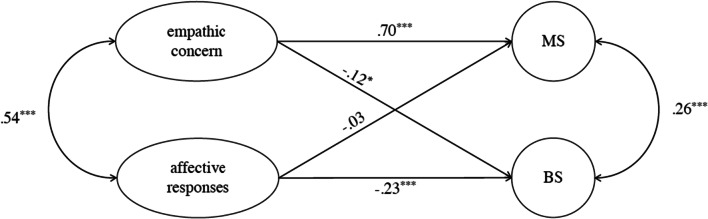
Standardized coefficients of the structural equation analysis (Model 1, Study 3). MS = moral sensitivity, BS = business sensitivity. * *p* < .05, *** *p* < .001

Figure 1 shows the standardized coefficients of Model 1. It suggests that empathic concern has a direct positive relation to MS (*β* = .70, *p* < .001), and a negative link to BS (*β* = −.12, *p* < .05). Affective responses, however, do not appear to have a significant relation to MS (*β* = −.03, *p* > .10), but a negative link to BS (*β* = −.23, *p* < .001). That is, the analyses mainly confirm H6b whereby empathic concern relates to MS and not to BS.

## General Discussion

Given that MS is the logical first step to ethical decision-making and therefore a central ability to manage moral decision-making(Bazerman & Tenbrunsel, 2011a; Miller et al., 2014; Rest, 1986), this work was motivated to contribute to the development of a solid measure of MS. As some authors stated, there is need for greater methodological rigor, since past measures have rarely tried to demonstrate reliability and construct validity beyond content validity (Jordan, 2007). Using heterogenous samples of employees (total *N* = 1168), the results of our investigations provide good evidence for the proposed structure, the reliability and validity of the vignette-based R-MSB, which has been developed to overcome some limitations of an earlier version of this measure (MSB measure) (Schmocker et al., 2019). EFAs and CFAs clearly supported a two-dimensional structure, revealing an MS and BS subscale with high internal consistency. Examining AVE and the square root of AVE, we also found strong support for the convergent and discriminant validity of MS and BS in reference to other related constructs. Further supporting the construct validity, we found that the MS and BS components were in most cases predictably associated with conceptually related constructs (such as moral intuitions, empathy, communal and agentic values, and Machiavellianism). Finally, the measure’s criterion-related validity was provided by demonstrating that diverse groups revealed different MS scores, while not differing in the BS scores. Specifically, employees expected to possess well-developed moral schemas (such as employees working in the educational, health or social sector) yielded higher MS scores than employees expected not to possess such dominant schemas (such as employees working in the trading, industry and construction sector).

### Strengths and Limitations

The R-MSB has several strengths. First, as a crucial difference from prior approaches, this measure allows to assess sensitivity for moral *and*business-related values. This is based on our claim that in practice, it would hardly be realistic and desirable that business professionals only pay attention to moral issues or pit moral against economic goals. On the contrary, modern organizations striving for sustainable and responsible business practices are faced with the challenge to find viable ways of reconciling ethical and economic demands (Geva, 2006; Zadek, 2007). Thus, professionals should ideally consider both ethical and economic standards. The R-MSB better accounts for the fact that individuals can be highly sensitive to *both* moral and business values, and that MS and BS do not necessarily have to be mutually exclusive.

Second, this measure was tested across large heterogenous samples of organizational employees, in two countries, and across a variety of economic sectors and jobs. This heterogeneity gives us some confidence as to the generalizability of the results, and that we can recommend the instrument for a broad use in the business world.

Third, compared to previous interview-based measures of MS and our earlier version of the measure, the adjusted instrument is simpler to use and generates data that can be analyzed more easily. Unlike the earlier version, the R-MSB assesses the recognition of moral and business-related issues and the ascription of importance to them in one rather than two steps. Thus, participants have to process each item only once and users of this instrument can easily average the different items to obtain the MS and BS scores. From a methodological point of view, the new version allows to conduct factor analyses (EFA, CFA) to gain insights about the underlying factor structure and the validity of the instrument.

An important avenue for future research would be to expand the R-MSB by affective and empathic components. Accounting for a common criticism that MS has mainly been studied in relation to cognitive processes (e.g., Clarkeburn, 2002; Jordan, 2007; Rest, 1986), our work does offer preliminary insight into the role of affective responses and empathic concern for MS. Several authors have stressed the importance of affective responses and empathic concern to have implications for MS (Narvaez, 2010; Tanner & Christen, 2014; Tenbrunsel & Smith-Crowe, 2008); yet, these relations have, to the best of our knowledge, not been empirically explored. Interestingly, in our study, only emphatic concerns appeared to affect MS directly. This supports the view that a sense of caring and relational connection to others, an understanding of how others might be affected by some actions, is helpful to facilitate MS. Interestingly, but unexpectedly, we found that affective responses had no direct link to MS but a negative one to BS. This may raise the question of putting the role of affective responsiveness in a somewhat different light. More precisely, affective responsiveness may represent reactions to tradeoffs between moral and economic values rather than direct reactions to violations of moral standards (Hanselmann & Tanner, 2008; Luce et al., 1997). In other words, affective responses may first of all reflect spontaneous and immediate aversions to possible ethical compromises. According to this view, reactions of outrage or anger are more likely to signal that potential ethical compromises would be seen as wrong and unjustified (Tetlock et al., 2000). As a consequence, when individuals treat ethical compromises as wrong and unjustified, they are also less sensitive to business issues. Of course, this could just be one possible interpretation of this result. Further studies are needed to improve our understanding of the connection between emotion, empathy and MS.

Furthermore, because affective processes are usually described as spontaneous processes (System 1), future studies may also include assessing reaction times to explore whether individuals respond, as expected, more quickly to affective than cognitive MS tasks. More research is also needed to examine how affective and empathic responsiveness can best be assessed and incorporated in a measurement of MS and BS.

Although this present research adds to the call for improved MS instrument development, it has also some limitations. Contrary to our expectations, MS was not significantly related to the established concept of moral attentiveness (Reynolds, 2008). This is somewhat surprising because our concept of MS is, at least conceptually, closely related to moral attentiveness. One possible explanation for this finding is that both approaches assess moral attentiveness on rather different levels of generality. While Reynold’s approach assesses moral attentiveness on a rather context-independent level, our measure applies a context-specific approach when assessing MS. According to Ajzen’s principle of compatibility (1988), however, two measures that assess the concept of interest at varying levels of generality or specificity are unlikely to correlate with each other.

The fact that our measure correlated with socially desirability scales needs to be discussed as well. Contrary to our expectations, we found that individuals high in MS were more likely to affirm positive personal qualities (*r* = .24, *p* < .01) and to reject negative ones (*r* = −.20, *p* < .01). While this may suggest that respondents were susceptible to a biased response style, we have seen, only recently, an increasing number of researchers proposing that social desirability scales may be likely to reflect truly social desirable traits rather than biased response styles (e.g., de Vries et al., 2014; Uziel, 2010). This new proposition is also based on the observation that individuals known to be highly committed to moral values such as honesty or integrity are also more likely to approve statements that describe themselves as individuals who avoid lies or fraud-statements which are usually associated with the positive pole of impression management scales. In line with this, a recent study by de Vries et al. (2014) found evidence for the association between social desirability, impression management and ethical traits (i.e., honesty-humility). For now, of course, we can only speculate whether a similar explanation may also account for our finding of MS being correlated with social desirability. Future research should attempt to provide more insights into this important topic.

Though our work has provided good evidence of the measure’s reliability and validity, further testing is needed. For example, it would be useful to test the measure’s test-retest and predictive validity in real situations to assure its full legitimacy. Finding that the measure is stable over time and correlates with morally sensitive behavior in the applied business context would add to the validity of this instrument. Furthermore, recall that we also found some support of the criterion validity of the R-MSB by comparing the MS and BS scores of two different employee groups. This finding is somewhat limited by the fact that those groups were extracted from the same samples based upon which also the other analyses (EFA, CFA) were conducted. Clearly, it would be useful, in a next step, to find further support for the criterion validity by examining groups of different samples.

### Implications for Theory and Practice

Over the past decades, researchers interested in better understanding unethical behavior in organizations have begun to shed more light on the role of moral awareness. Since the pioneering work by Rest (1986), MS is seen as the first step and the foundation of ethical decision-making and behavior (Miller et al., 2014). Without recognizing that a moral issue is at stake, it is unlikely that a decision-maker will account for and act upon it (Clarkeburn, 2002; Rest, 1986; Sparks & Hunt, 1998). Yet, despite the relevance of MS, its impact on decision-making and on behavior is empirically rather unexplored. Thus, we believe that researchers can benefit from applying our instrument to empirically investigate the relations between MS and related constructs. It is not only the link between MS, decision-making and behavior which awaits empirical examination, the instrument can be used to investigate situational and individual differences and how they may affect MS. One question of interest is: Are morally sensitive individuals less or even more likely to disengage from moral standards to avoid self-sanctions (moral disengagement)? Given that our instrument assesses sensitivity for moral and business-related issues, an obvious question that arise is: Are leaders or employees that are aware of *both* issues more likely to suffer more from conflicts? Further, it would be interesting to find answers to questions such as: How does moral sensitivity change in the course of socialization into an organization? Is career progression positively or negatively related to MS? Are morally sensitive individuals more resistant to “ethical fading” (Tenbrunsel & Messick, 2004) and socialization into unethical practices?

Furthermore, we believe that organizations and business leaders can benefit from applying the perspective to MS to their company. Our measure can be used to identify levels of MS and potential ethical risks. Furthermore, it can be used to assess new personnel or to evaluate trainings or other interventions that were applied to enhance MS. As Miller et al. (2014) noted “understanding levels of moral awareness and how they are affected by trainings may help organizations avert problems and even catastrophes due to lack of awareness about moral issues” (p. 37). Of course, MS does not suffice to avoid unethical behavior, but a necessary “point of departure” whereby moral issues and behavior will be accounted for into the process of judgment and decision-making (Jordan, 2009; Tenbrunsel & Smith-Crowe, 2008).

Finally, in terms of intervention strategies to support MS, our findings do suggest that encouraging empathic responsiveness may be a viable way of enhancing MS. Research has demonstrated that encouraging individuals to put oneself in other people’s place can reinforce learning and retention (e.g., Feshbach & Feshbach, 2009). That is, promoting MS by encouraging empathic responsiveness is also interesting from an educational perspective.

## Conclusion

To conclude, the results reported here support the use of the R-MSB as a relatively effective and psychometrically sound measure of MS and BS. Moral sensitivity is imperative for dealing with ethical challenges and misconduct. We believe that the R-MSB has the potential to contribute to new and interesting research that is of value for research and practice alike. Among other things, the measure offers a basis for examining antecedents and consequences of MS and BS, for giving feedback to employees and leaders about their current state of sensitivity, and for inferring and monitoring targeted interventions to improve MS.

## Supplementary Information

ESM 1 (DOCX 28 kb)

## Data Availability

The datasets generated during and/or analysed during the current study are available in the Open Science Framework repository, https://osf.io/tdyau/?view_only=46d9d548faa1416e94143c7fda1acf06.
