# Supplementary material for: Moral sensitivity in business: A revised measure
Source: Curr Psychol. 2021 Sep 23;42(12):10277–91. doi: 10.1007/s12144-021-01926-x (PMC10195703; doi:10.1007/s12144-021-01926-x)
Supplement: Supplementary file 1 — (DOCX 28 kb) [file 12144_2021_1926_MOESM1_ESM.docx]

**Appendix**

**Vignette Example (original in German)**

Prime Financial Industries Inc. (PFI) has decided to strengthen its external communication resources in order to respond appropriately to the current tight market situation. The newly advertised position in the communications department has received numerous promising applications by the deadline. After extensive analyses, the personnel department was finally able to reduce the number of potential candidates to two.

Prior to the start of the final selection round, an enquiry was received from a long-standing client: He had wished for his son to join the company for quite some time. An examination of the dossier submitted shows that the son has sufficient qualifications for the position to be filled. The personnel department is now unsure how to react.

**Value Statements relating to Vignette**

How important is it to you to consider whether the pending decision…

... could make other applicants not feel appreciated or not? (= Moral Value (MV), Respect)

... could harm the other applicants or not? (= MV, Non-Maleficience)

... could impair the loyalty of other internal applicants to the company or not? (= MV Loyalty)

... cold disadvantage other applicants or not? (= MV, Fairness)

... could worsen the company’s performance or not? (= Business Value (BV), Performance)

... could worsen the company’s profit or not? (= BV, Profitability)

... cold worsen the public image of PFI or not? (= BV, Reputation)

... could result in a deterioration of the company’s positioning or not? (= BV, Competition)

**Items assessing affective reactions** (adapted to the respective vignette)

In case the customer's son would be chosen for the position, I would find that ...

... outrageous

... embarrassing

... acceptable (R)

... praiseworthy (R)

**Items assessing empathic concern (adapted to the respective vignette)**

To see how someone might be hired here because of personal relationships, ...

... triggers compassion in me for all those who have applied in vain.

... affects me also as an unaffected person very much.

... makes me want to protect the other applicants.

... I consider it to be a purposeful step for the company. (Filler item)

... I consider to be unavoidable for the company. (Filler item)
